# Editorial: Influenza and related viruses: Epidemiology, pathogenesis, and therapeutics

**DOI:** 10.3389/fmolb.2022.1117067

**Published:** 2023-01-04

**Authors:** Kumari Asha, Clement Meseko, Binod Kumar

**Affiliations:** ^1^ Department of Microbiology and Immunology, Chicago Medical School, Rosalind Franklin University of Medicine and Science, North Chicago, IL, United States; ^2^ Regional Centre for Animal Influenza, National Veterinary Research Institute, Vom, Nigeria; ^3^ Department of Antiviral Research, Institute of Advanced Virology, Thiruvananthapuram, Kerala, India

**Keywords:** Influenza A virus, Influenza, SARS-CoV2, Coronavirus, COVID-19, Computational approach

Influenza is a highly contagious respiratory disease caused by influenza A, B, and C viruses in humans and accounts for 30% of all respiratory viral infections. These viruses (influenza A to a greater extent) are responsible for annual seasonal epidemics of variable severity in people across all ages around the world (Kumar et al., 2018). Influenza A viruses of zoonotic origin, in particular, have been responsible for several past pandemic with a significant impact on human health and killing millions of people across the globe (Khanna et al., 2009). As per WHO, every year, an estimated 650,000 deaths are attributed to respiratory diseases linked to seasonal influenza alone. Similarly, SARS-CoV2, another virus of zoonotic origin, has been the cause of ongoing pandemic leading to a significant rate of morbidity and mortality. The COVID-19 pandemic has surpassed all previous pandemics caused by viruses in geographical spread raising concerns on our preparedness measures and plans at global levels and teaching hard lessons for future preparedness. As of 2 December 2022, a total of 6,618,579 deaths have been reported to WHO (2022). Similarly, RSV infections, traditionally considered less severe than influenza, have raised recent concerns because of increased outbreaks and linked hospitalizations in young children and adults (Bardsley et al., 2022; Busack and Shorr, 2022). It is a common cause of lower respiratory tract infections in older adults and young children. Likewise, 80 different adenovirus types are known to infect humans causing acute clinical manifestations or often persist under the host immune responses causing significant loss of health and life in high-risk individuals.

The Research Topic covered in this Research Topic provides useful insights into some of the very important aspects of respiratory viruses. Sobitan et al. demonstrated the usage of computational approach to perform saturation mutagenesis of the spike protein of SARS-CoV1 to identify the residues important for its functions. The article showed the effect of missense mutations in the spike stability and binding affinity with ACE receptor using structure-based energy calculations and concluded that there are structural similarities between SARS-CoV1 and SARS-CoV2 S proteins and that target mutations on the S generate similar effects on their stabilities between both CoVs (Sobitan et al.). Such findings may be very helpful in designing antiviral drugs against both SARS-CoV1 and SARS-CoV2.

Another study by Hassan et al. also utilized the computational approach to predict the site directed miRNAs as important players of transcriptional regulators against Influenza C virus (ICV) infection. ICV causes upper respiratory tract infections such as cold, pharyngitis and laryngotracheitis. The authors used several platforms such as RNA22, RNAhybrid and miRanda to predict the miRNAs playing crucial role in host response to influenza C virus infection. All the proteins of ICV were subjected to analysis and it was concluded that the HEF of ICV had the highest prevalence as a potential miRNA target leading path to discovery of potential therapeutic intervention against ICV (Hassan et al.).

Hoter and Naim studied the impact of continuous spike (S1 mutations) on the pathogenicity of SARS-CoV2. They analyzed the biosynthesis and secretion of the RBD double mutants L452R and E484Q present in the Indian B.1.617 variant compared to the wildtype RBD in mammalian cells. Their findings showed that these double RBD mutants resulted in higher expression level and secretion of spike S1 protein than other mutations and further showed enhanced interaction with ACE2 receptor. This study reiterates the impact of evolving spike mutations on the pathogenicity of SARS-CoV2 (Hoter and Naim).

Adenovirus is one of the common respiratory viruses causing mild to severe infections with symptoms very similar to common cold or flu. Mao et al. in their case report, discussed an unusual multiple embolism in a patient with adenovirus pneumoniae. The patient was diagnosed to be infected with the Human adenovirus B strain 55 as per genome sequence similarity. The authors discussed that thrombosis associated with adenovirus pneumoniae is extremely rare, especially among the immunocompetent adults and hypothesized that it may be related to direct viral endothelial injury and procoagulant activity. The authors emphasized that more attention should be given to unusual extrapulmonary manifestations of adenovirus infection and specially the D-dimer levels may be indicative of thrombotic events in such patients and may be used as an indicator for early identification and timely intervention (Mao et al.).

Overall, this Research Topic shed light on the advancement of approaches that is required for early identification of viral infections, associated markers and possible therapeutic interventions. While it is imperative to have good understanding of the virology behind infections, it is all the more important to identify predictive and early markers that can help in interventions to reduce hospitalizations and death. Computational approaches have shown tremendous potential in providing early clues to design and develop antiviral strategies and manage emerging and re-emerging respiratory viral infections.
